# Predictive model for identification of gangrenous or perforated appendicitis in adults: a multicenter retrospective study

**DOI:** 10.1186/s12876-024-03445-y

**Published:** 2024-10-09

**Authors:** Yun Liang, Maimaitiaili Sailai, Rui Ding, Baihitiyaer Yimamu, Tayierjiang kazi, Ming He, Zehui Liu, Junyu Lin, Yile Liu, Chaolun Deng, Jiangtao Huang, Xingwei Zhang, Zheng Chen, Yonghui Su

**Affiliations:** 1 Department of Gastrointestinal Surgery, The Fifth Affiliated Hospital of Sun Yat- Sen University, No.52 Mei Hua East Road, Xiang Zhou District, Zhuhai, Guangdong 519000 China; 2 Department of Colorectal Surgery, The Sixth Affiliated Hospital, Sun Yat-sen University, Guangzhou, Guangdong 510655 China; 3 Department of General Surgery, The First People’s Hospital of Kashagr, Xinjiang Uygur Autonomous Region, Kashagr Region, 844000 China

**Keywords:** Gangrenous/perforated appendicitis, Nomogram, Risk factor, Prediction

## Abstract

**Background:**

Gangrene and perforation are severe complications of acute appendicitis, associated with a higher mortality rate compared to uncomplicated appendicitis. Accurate preoperative identification of Gangrenous or perforated appendicitis (GPA) is crucial for timely surgical intervention.

**Methods:**

This retrospective multicenter study includes 796 patients who underwent appendectomy. Univariate and multivariate logistic regression analyses are used to develop a nomogram model for predicting GPA based on laboratory tests and computed tomography (CT) findings. The model is validated using an external dataset.

**Results:**

Seven independent predictors were included in the nomogram: white blood cell count, lymphocyte count, D-dimer, serum glucose, albumin, maximum outer diameter of the appendix, and presence of appendiceal fecalith. The nomogram achieved good discrimination and calibration in both the training and testing sets. In the training set, the AUC was 0.806 (95%CI: 0.763–0.849), and the sensitivity and specificity were 82.1% and 66.9%, respectively. The Hosmer-Lemeshow test showed good calibration (*P* = 0.7378). In the testing set, the AUC was 0.799 (95%CI: 0.741–0.856), and the sensitivity and specificity were 70.5% and 75.3%, respectively. Decision curve analysis (DCA) confirmed the clinical utility of the nomogram.

**Conclusion:**

The laboratory test-CT nomogram model can effectively identify GPA patients, aiding in surgical decision-making and improving patient outcomes.

## Background

Acute appendicitis (AA) is a common surgical emergency globally, with an annual incidence ranging from 96.5 to 100 cases per 100,000 adults. It predominantly affects individuals aged 20 to 40 years, with a lifetime risk of 7–8% in the general population [1]. AA can be categorized into simple and complex appendicitis, with the latter characterized by appendiceal inflammation involving gangrene, perforation, or abscess formation. In the United States, complex appendicitis represents approximately 30% of all acute appendicitis cases [2]. Complex appendicitis poses a higher risk of complications such as diffuse peritonitis and septic shock, leading to increased mortality rates compared to uncomplicated appendicitis [3].

The standard treatment approach for clinically diagnosed AA typically involves appendectomy with the addition of antibiotics. Laparoscopic appendectomy has become the preferred surgical technique [3], yet concerns regarding surgical complications, anesthesia risks, and financial burdens have prompted clinicians to explore alternative treatment strategies that prioritize patient well-being. Additionally, the occurrence of negative postoperative pathology results for appendicitis in some patients who underwent appendectomy underscores the need for more precise clinical decision-making [4].

The management of appendicitis has garnered increasing attention, with studies demonstrating the efficacy and safety of antibiotic therapy alone for uncomplicated appendicitis (UA) [5–8]. While a conservative approach followed by appendectomy has gained prominence in managing appendicitis at our institution, it is essential to recognize that antibiotic treatment for cases of gangrenous or perforated appendicitis (GPA) carries a heightened risk of failure, potentially leading to severe complications such as peritonitis and septic shock [1, 9, 10]. Timely appendectomy remains the optimal strategy for GPA cases, as failure of non-surgical treatment often necessitates open surgery, hemicolectomy, and prolonged hospitalization [11, 12]. Therefore, early identification of GPA patients within the appendicitis cohort is crucial for surgeons to make well-informed decisions regarding surgical intervention.

Recent literature has introduced scoring models for predicting complex acute appendicitis; however, these models often lack objectivity and repeatability due to limited variables. In addition, the amount of patient data included at the same time is relatively small or only data from a single medical center is used, and the constructed model has not been further tested [13–19]. Hence, this study aims to develop a novel scoring model incorporating radiographic findings, laboratory results, and clinical parameters to predict GPA (excluding appendicitis with abscesses) and aid clinicians in optimizing treatment decisions for appendicitis patients.

## Methods

### Patients enrollment

We conducted a retrospective analysis involving 937 patients who underwent appendectomy at the Fifth Affiliated Hospital of Sun Yat-sen University and the First People’s Hospital of Kashgar Region between January 2022 and December 2023. All enrolled patients underwent non-contrast CT scans of the lower abdomen or the entire abdomen, and all appendices were subjected to pathological diagnosis following surgical resection. Decisions regarding patient care and surgical scheduling at the medical center were primarily made by doctors in the medical center. This study focused on adult cases of acute appendicitis and excluded patients meeting any of the following criteria: (I) Age over 65 or under 18 (*n* = 71); (II) Other acute abdominal conditions or pregnancy (*n* = 10); (III) Appendectomy leading to right hemicolectomy (*n* = 3); (IV) Non-inflammatory appendix diseases (*n* = 1); (V) Appendix abscess (*n* = 14); (VI) Incomplete clinical data (*n* = 42). Ultimately, 796 patients were included in the study.

**Fig. 1:**
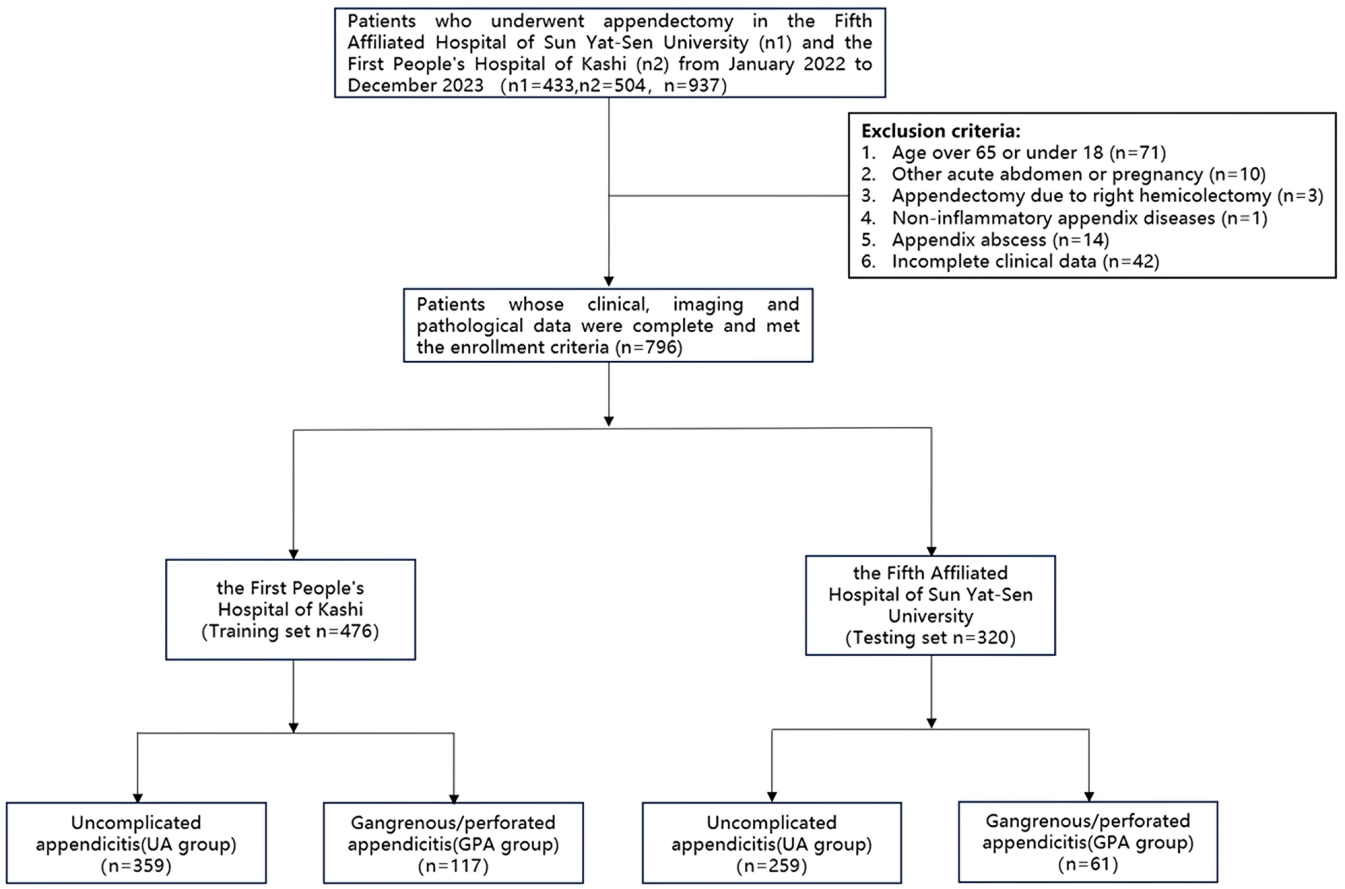
Flowchart of patient selection and exclusion process

The First People’s Hospital of Kashgar Region was designated as the training group, while the Fifth Affiliated Hospital of Sun Yat-sen University served as the testing group. We will conduct a logistic regression analysis using the training cohort to develop a prediction model, and then we will use the validation set for external validation to assess the model’s performance. The patients were divided into two groups: GPA group and UA group. The GPA group will consist of perforated and gangrenous appendicitis, whereas the UA group will comprise simple appendicitis and suppurative appendicitis. Perforated appendicitis will be identified based on pathological diagnosis or surgical findings, while the other types of appendicitis will be confirmed through pathological diagnosis. The patient recruitment process is outlined in Fig. 1. The Institutional Review Board of the Fifth Affiliated Hospital of Sun Yat-sen University and the First People’s Hospital of Kashgar Region approved this retrospective study and waived the requirement for obtaining informed consent from the patients.

### Study variables

The medical center’s electronic medical records were queried for patient data, laboratory results, and abdominal CT scan images. Patient characteristics included gender, age, duration of abdominal pain, presence of diabetes and hypertension, history of abdominal surgery, history of preoperative antibiotic therapy, and body mass index (BMI). Collect and document the results of the patient’s most recent blood test conducted prior to surgery. Laboratory tests encompassed white blood cell count (WBC), neutrophil count, lymphocyte count, neutrophil-to-lymphocyte ratio (NLR), platelet count (Plt), mean platelet volume (MPV), platelet distribution width (PDW), fibrinogen level (Fg), D-dimer level (D-D), procalcitonin level (PCT), total bilirubin level (Tbil), glucose level (Glu), and albumin level (Alb). Additionally, results from abdominal CT scans included the presence of appendiceal fecalith, the presence of peri appendiceal fat stranding, the presence of free peritoneal fluid and the maximal diameter of the appendix (mm). These variables are highly objective and readily obtainable from hospitals of all levels.

### Laboratory CT nomogram construction and performance assessment

Predictive factors, including patient characteristics, laboratory tests, and CT reports, were assessed using a logistic regression algorithm within the training set. All variables that showed significant significance (*P* < 0.05) in the univariate analysis were included in the subsequent multivariate logistic regression analysis. Independent predictive factors were selected to develop a predictive model. Subsequently, we created a laboratory-CT nomogram based on the findings from the multivariate logistic regression.

The performance of the laboratory-CT nomogram was evaluated in terms of its discrimination and calibration within the training set. The AUC of the receiver operating characteristic (ROC) was used to assess the model’s discrimination. Calibration was evaluated using a calibration curve and the Hosmer-Lemeshow test to gauge the goodness-of-fit of the nomogram. Finally, decision curve analysis (DCA) was conducted to assess the clinical utility of the nomogram by calculating net benefits at various threshold probabilities.

### Validation of the laboratory-CT nomogram

External validation of the laboratory-CT nomogram was conducted using data from the Fifth Affiliated Hospital of Sun Yat-sen University. The logistic regression formula developed in the training set was applied to all patients in the validation set. Subsequently, the performance of the nomogram was assessed by evaluating its calibration (using a calibration curve), discrimination (through AUC), and clinical utility (via DCA).

### Statistical analyses

All statistical analyses were conducted using the R statistical software, version 3.5.1 (R Foundation for Statistical Computing; https://www.r-project.org/). Continuous variables were compared using the Mann–Whitney U test, while categorical variables were compared using the chi-square test. A significance level of *P* < 0.05 was considered statistically significant. Logistic regression analysis, nomogram development, calibration plots, ROC curve analysis, The Hosmer-Lemeshow test, and decision curve analysis (DCA) were performed using the respective R packages. All statistical tests were two-tailed, and *P* < 0.05 was considered statistically significant.

## Results

### Characteristics of the training and testing cohort

A total of 798 patients were included in the final analysis. Of these, 476 patients from the first medical center were allocated to the training set, while 320 patients from the second medical center constituted the validation set. The characteristics and CT scan reports of patients with AA in both the training and validation sets are summarized in Table 1. The incidence of GPA in the two groups was well balanced, with rates of 24.6% in the training set and 19.1% in the validation set (*P* = 0.081).

**Table 1:** Comparison between the training cohort and testing cohort

	Training cohort	Testing cohort	*P*
**Patient characteristics**			
Gender			0.168
Male	285(59.9%)	175(54.7%)	
Female	191(40.1%)	145(45.3%)	
Age, years	36.00(18.00–65.00)	37.50(18.00–65.00)	0.384
Duration of abdominal pain (h)	24.00(1.00-240.00)	24.00(3.00-240.00)	˂ 0.001
Hypertension			
Yes	30(6.3%)	10(3.1%)	0.065
No	446(93.7%)	310(96.9%)	
Diabetes			0.131
Yes	13(2.7%)	3(0.9%)	
No	463(97.3%)	317(99.1%)	
History of antibiotic treatment before surgery			˂ 0.001
Yes	24(5.0%)	66(20.6%)	
No	452(95.0%)	254(79.4%)	
Previous history of abdominal surgery			0.023
Yes	81(17.0%)	35(10.9%)	
No	395(83.0%)	285(89.1%)	
BMI (kg/m2)	23.88(14.69-50.00)	23.13(14.69–34.94)	˂ 0.001
**Laboratory findings**			
WBC (×10^9/L)	11.96(2.57–61.58)	12.44(3.90-26.45)	0.26
Neutrophil (×10^9/L)	10.19(1.45–38.69)	10.17(1.25–25.20)	0.696
Lymphocyte (×10^9/L)	1.32(0.18–37.30)	1.42(0.28–4.07)	0.002
NLR	6.62(0.43–82.30)	8.37(1.82–52.90)	˂ 0.001
Plt (×10^9/L)	240.00(3.70–850.00)	227.50(62.00-434.00)	0.042
MPV (fL)	9.30(0.36–89.20)	9.50(6.70–905.00)	0.003
PDW (fL)	16.10(0.54-39.00)	16.10(8.60–18.10)	0.972
Fg (g/L)	3.90(0.19–114.90)	3.46(1.34–12.09)	0.003
D-D (µg/ml)	0.52(0.04–69.48)	0.30(0.03-148.43)	˂ 0.001
PCT (ng/ml)	0.12(0.02–100.00)	0.08(0.05–50.62)	0.027
Tbil (µmol/L)	14.15(2.60–513.00)	15.15(3.20-101.60)	0.071
Glu (mmol/L)	4.60(1.60–30.00)	5.90(3.30-14.06)	˂ 0.001
Alb (g/L)	40.90(10.10–93.20)	44.50(30.40–64.00)	˂ 0.001
**CT findings**			
The maximal diameter of the appendix (mm)	11.00(0.00–40.00)	11.00(6.00–25.00)	0.433
Presence of fecalith			˂ 0.001
Yes	380(79.8%)	136(42.5%)	
No	96(20.2%)	184(57.5%)	
Presence of peri appendiceal fat stranding			0.168
Yes	354(74.4%)	239(74.7%)	
No	122(25.6%)	81(25.3%)	
Presence of free peritoneal fluid			˂ 0.001
Yes	160(33.6%)	36(`11.2%)	
No	316(66.4%)	284(88.8%)	
**Acute appendicitis**			0.081
GPA	117(24.6%)	61(19.1%)	
UA	359(75.4%)	259(80.9%)	

(Categorical data is presented as percentages, while continuous data is presented as median (min-max)

### Comparisons between GPA and UA groups using univariate and multivariate analyses in the training cohort

Univariate analysis was used to compare the GPA group and the UA group in the training set. Table 2 presented patient characteristics of the two groups, showing no statistically significant differences in age, gender, and duration of abdominal pain. Laboratory tests revealed significantly higher levels of WBC (*P* < 0.001), neutrophils (*P* < 0.001), NLR (*P* < 0.001), PCT (*P* < 0.001), D-dimer (*P* < 0.001), and Glucose (*P* = 0.006) in the GPA group compared to the UA group. Conversely, lymphocyte and albumin levels were higher in the UA group. CT scans showed that the maximum diameter of the appendix (*P* < 0.001) and the presence of appendicular fecalith (*P* = 0.046) were significantly greater in the GPA group than in the UA group. A multivariate logistic regression analysis was performed on the variables mentioned (WBC, neutrophils, lymphocytes, NLR, PCT, D-dimer, Glucose, Albumin, maximum diameter of the appendix, and presence of fecalith). The results indicated that WBC, lymphocytes, D-dimer, Glucose, Albumin, maximum diameter of the appendix, and presence of fecalith were significantly associated with GPA and acted as independent predictors (Table 2).

**Table 2:** Univariate and Multivariate Logistic Regression Analysis of the predictors for GPA

Variable	Univariate analysis		Multivariate analysis	
	OR (95% CI)	*P*	OR (95% CI)	*P*
Age (years)	1.015(0.998-1.032)	0.077	-	-
Male SEX	0.908(0.594-1.387)	0.656	-	-
Duration of abdominal pain	1.002(0.999-1.004)	0.214	-	-
Hypertension	1.124(0.487-2.598)	0.784	-	-
Diabetes	0.550(0.120-2.519)	0.441	-	-
History of antibiotic treatment	1.911(0.813-4.490)	0.137	-	-
Previous history of abdominal surgery	0.653(0.357-1.195)	0.167	-	-
BMI (kg/m^2^)	1.011(0.962-1.062)	0.67	-	-
WBC (×10^9/L)	1.094(1.046-1.145)	<0.001	1.284(1.001-1.647)	0.049
Neutrophil (×10^9/L)	1.128(1.075-1.183)	<0.001	0.894(0.693-1.154)	0.39
Lymphocyte (×10^9/L)	0.388(0.266-0.567)	<0.001	0.284(0.141-0.574)	<0.001
NLR	1.053(1.028-1.080)	<0.001	0.969(0.930-1.010)	0.138
Plt (×10^9/L)	0.999(0.997-1.002)	0.619	-	-
MPV (fL)	1.015(0.981-1.051)	0.387	-	-
PDW (fL)	1.034(0.920-1.162)	0.572	-	-
Fg (g/L)	1.018 (0.982-1.056)	0.334	-	-
D-D (μg/ml)	1.284(1.160-1.422)	<0.001	1.136(1.009-1.278)	0.035
PCT (ng/ml)	1.059(1.030-1.088)	<0.001	1.014(0.984-1.044)	0.362
Tbil (μmol/L)	1.006(0.997-1.015)	0.178	-	-
Glu (mmol/L)	1.164(1.046-1.296)	0.006	1.167(1.045-1.303)	0.006
Alb (g/L)	0.921(0.888-0.955)	<0.001	0.956(0.917-0.997)	0.035
Maximal diameter of the appendix (mm)	1.081(1.038-1.124)	<0.001	1.086(1.038-1.136)	<0.001
Presence of fecalith	1.810(1.010-3.243)	0.046	2.387(1.181-4.826)	0.015
Presence of peri appendiceal fat stranding	1.667(0.993-2.799)	0.053	-	-
Presence of free peritoneal fluid	1.263(0.818-1.950)	0.293	-	-

### Laboratory-CT nomogram construction and performance assessment

**Fig. 2:**
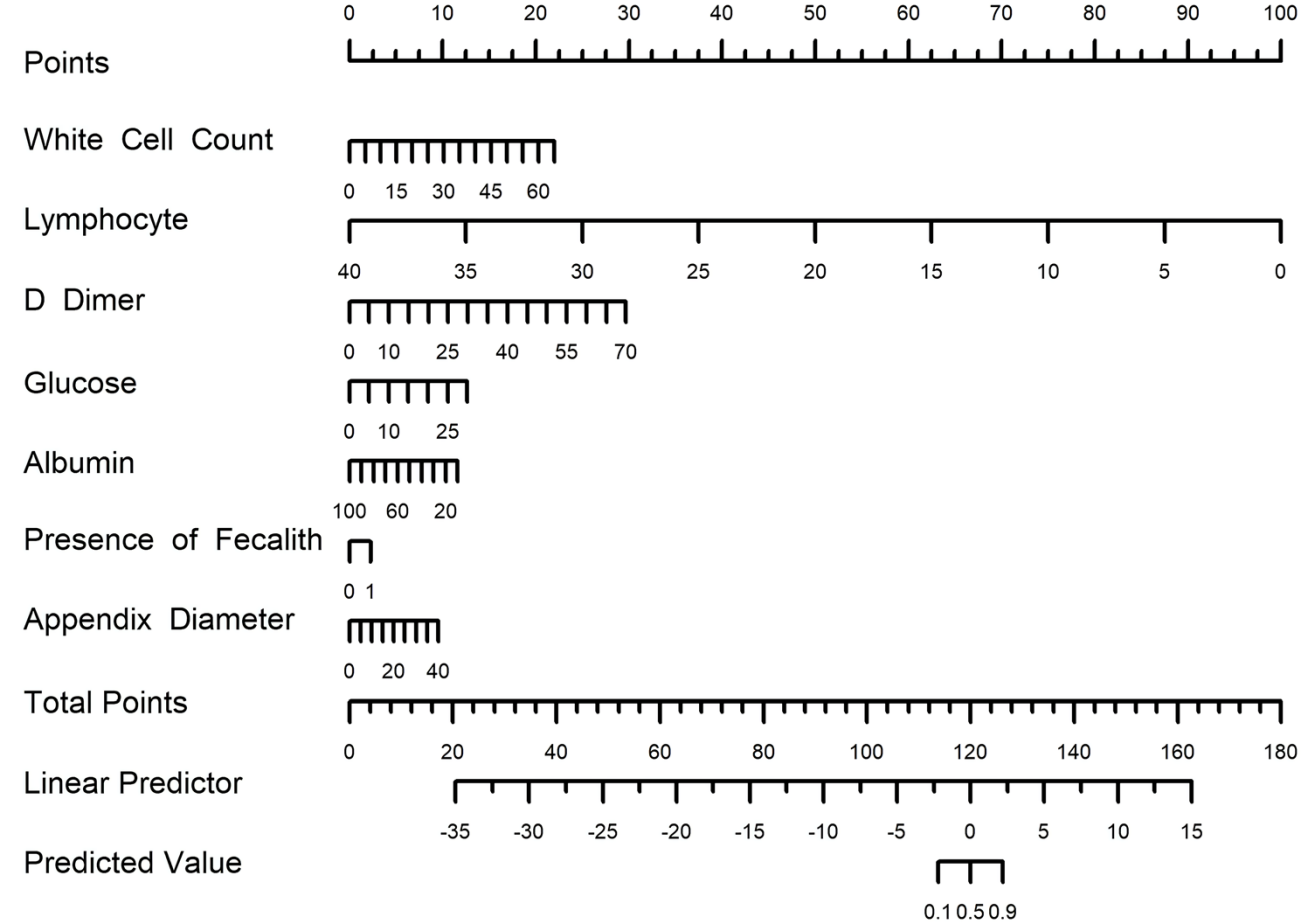
Laboratory-CT nomogram for predicting the probability of GPA in patients with appendicitis. (To use the nomogram, locate your patient’s risk factors, draw lines from their values to the “Points” axis, sum the points, then draw a line down from the total points to find the predicted outcome on the “Predicted Value” axis. For binary variables, 0 represents “no” and 1 represents “yes”. And Appendix diameter means the maximal outer diameter of the appendix.)

**Fig. 3:**
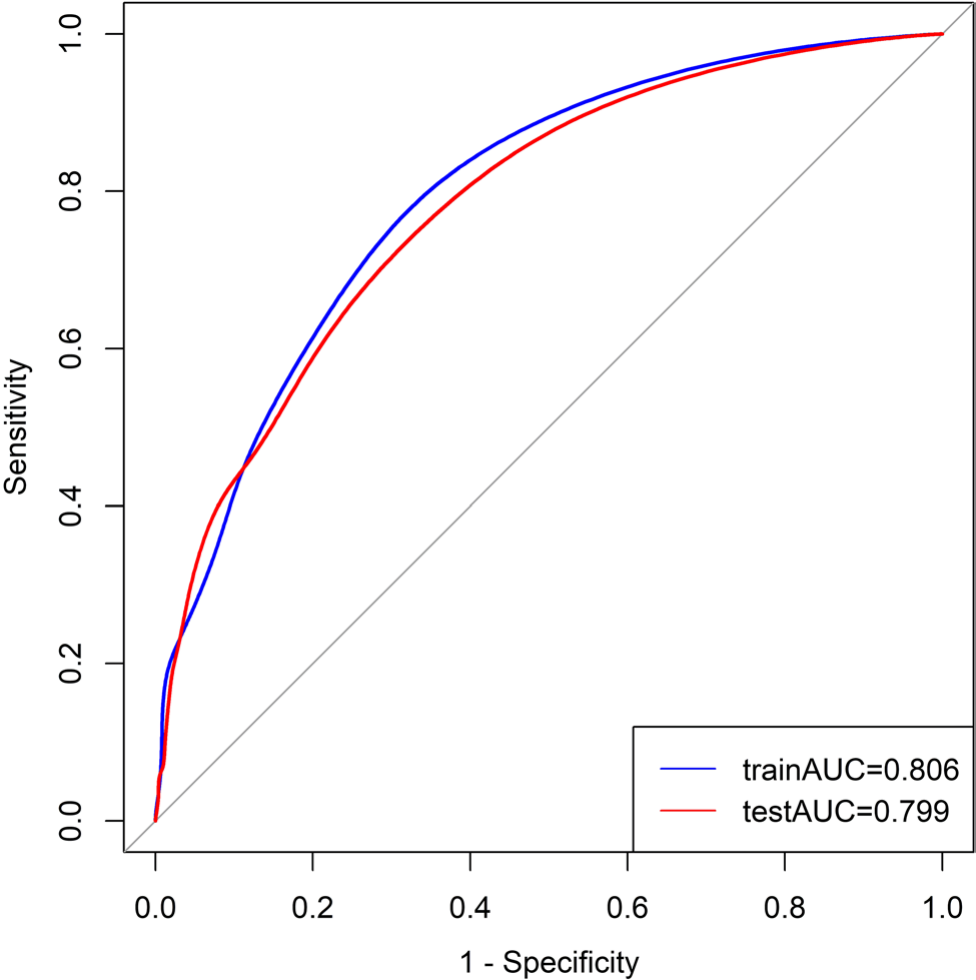
Receiver operator characteristic curve of the laboratory-CT nomogram in the training set and testing set

**Fig. 4:**
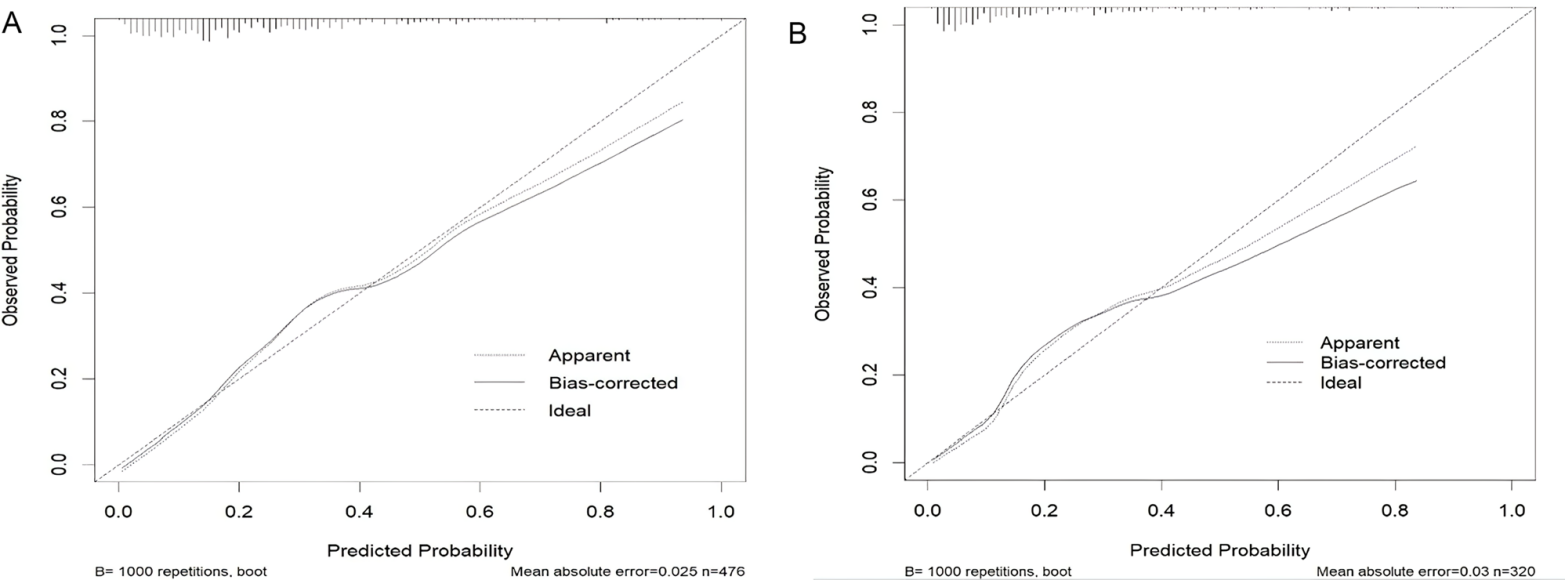
Calibration curve of the laboratory-CT nomogram prediction in the cohort. **A** Calibration curves of training set. **B** Calibration curves of testing set

**Fig. 5:**
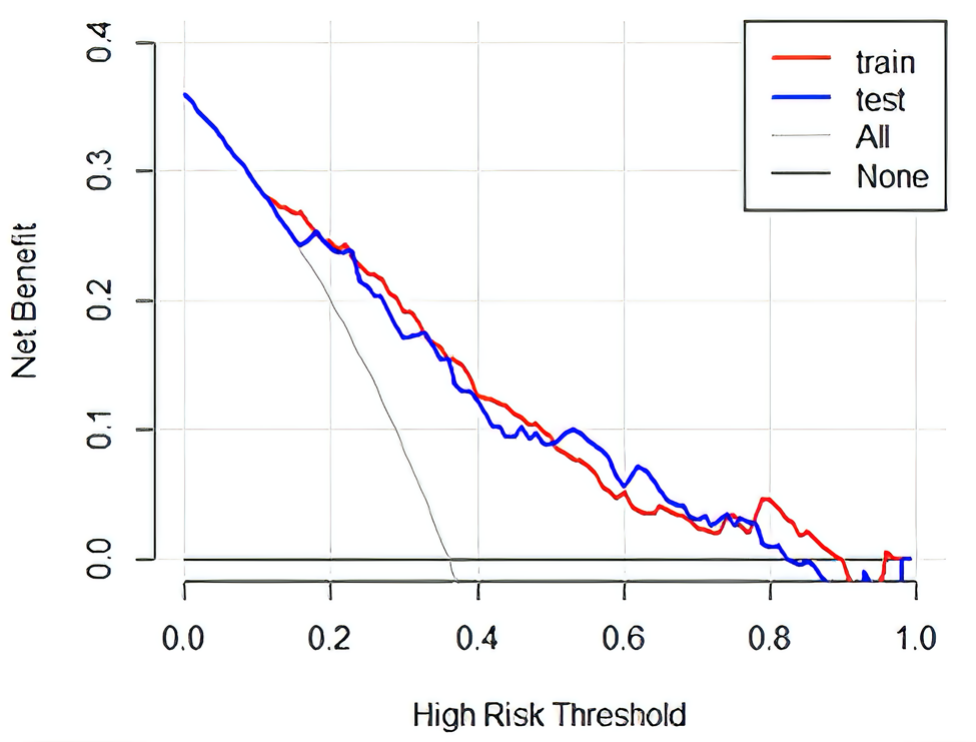
The decision curve analysis for the laboratory-CT nomogram in the training set and the testing set

A nomogram was developed based on laboratory results and abdominal CT findings by incorporating the seven predictors identified in the logistic regression analysis described earlier (Fig. 2). The nomogram exhibited a strong discriminatory ability in the training set, with an AUC of 0.806 (95% CI: 0.763–0.849) (Fig. 3). In Table 3, the model’s accuracy in the training set was 70.6%, with a sensitivity of 82.1%and specificity of 66.9%. The positive predictive value was 44.7%, and the negative predictive value was 92.0%. The calibration curve showed that the model’s predictions closely matched the actual observations in the training set (Fig. 4A). Moreover, the p-value of the Hosmer-Lemeshow test was 0.7378, indicating satisfactory calibration. The results of decision curve analysis (DCA) in the training set are depicted in Fig. 5. The DCA curve illustrated that utilizing the laboratory-CT nomogram for decision-making on GPA and emergency surgery can lead to a greater net benefit under certain circumstances. Consequently, our nomogram emphasizes its importance as a valuable tool for clinical decision-making.

**Table 3:** Predictive value of the Laboratory-CT nomogram

Laboratory-CT nomogram	AUC (95% CI)	ACC	SEN	SPE	PPV	NPV
training set	0.806(0.763–0.849)	0.706	0.821	0.669	0.447	0.920
testing set	0.799(0.741–0.856)	0.744	0.705	0.753	0.402	0.915

(ACC: Accuracy; SEN: Sensitivity; SPE: Specificity; PPV: Positive Predictive Value; NPV: Negative Predictive Value)

### Validation of the laboratory-CT nomogram

Using patient data from the Fifth Affiliated Hospital of Sun Yat-Sen University for external validation, the nomogram also exhibited satisfactory discriminant ability with an AUC value of 0.799 (95% CI: 0.741–0.856) in the testing set. The calibration in the validation set was considered good (Fig. 4B), as evidenced by a P value of 0.3101 from the Hosmer-Lemeshow test, indicating a favorable fit. Additionally, the DCA curve indicated that the lab-CT nomogram yielded a higher net benefit in the validation set within a specific threshold range (Fig. 5).

## Discussion

In this study, we developed and validated a novel scoring model for GPA in adults using a combination of patient clinical characteristics, laboratory tests, and abdominal CT results. Univariate and multivariate logistic regression analyses identified seven independent predictors of GPA: WBC, lymphocyte, D-dimer, glucose, and albumin levels from laboratory tests, as well as the maximum outer diameter of the appendix and the presence of appendix fecalith as detected through abdominal CT scans. Meanwhile, parameters such as fibrinogen (Fg) were excluded due to their lack of statistical significance in the univariate analysis. Similarly, factors like neutrophil-to-lymphocyte ratio (NLR) and procalcitonin (PCT) were excluded from the final model due to their lack of statistical significance in the multivariate analysis. A nomogram was constructed and validated using these predictors to assess the risk of GPA in adults. The laboratory-CT model exhibited strong diagnostic accuracy in predicting the occurrence of gangrenous and perforated appendicitis in adults.

Acute appendicitis is a common abdominal emergency in adults worldwide, often requiring appendectomy as a surgical intervention [1]. Traditionally, surgeons have relied on clinical symptoms, laboratory findings (such as elevated white blood cell count), and abdominal CT scans to guide the decision-making process for surgical intervention in affected patients. Recent research has demonstrated the efficacy of antibiotic therapy for uncomplicated appendicitis, while complicated cases typically still require appendectomy [3–9]. Accurately identifying complicated appendicitis is crucial for guiding appropriate treatment decisions and avoiding unnecessary urgent surgeries. However, there is limited literature on predictive models using laboratory tests and abdominal CT scans to anticipate complicated appendicitis in adult patients.

Previous studies have reported various indicators associated with GPA [20–31]. WBC is commonly used as a blood marker for diagnosing appendicitis [21, 22], while lymphocyte levels are inversely correlated with the severity of appendicitis inflammation, typically reduced in cases of gangrenous appendicitis [23]. Our study confirmed WBC and lymphocytes as independent predictors of GPA, consistent with existing literature.

Research has indicated a bidirectional relationship between the coagulation system and inflammatory response [24]. D-dimer, a degradation product of fibrin, has been studied in the context of non-traumatic acute abdomen, suggesting its potential to replace white blood cells in diagnosing this condition [25]. Furthermore, Blunt et al. found that D-dimer demonstrates high specificity but low sensitivity in diagnosing appendicitis [25]. Previous studies have also suggested a potential link between D-dimer levels and the severity of appendicitis [26]. Elevated blood sugar levels have been noted as a response to inflammatory triggers and physical stress, creating a favorable environment for bacterial growth and worsening inflammation in the appendix [27]. Additionally, albumin (ALB), a negative acute-phase reactant produced by the liver, decreases in response to inflammation, with implications for inflammation severity, disease prognosis, and mortality [28]. In our research, these markers were identified as independent predictors of GPA through multivariate analysis, supporting the pathophysiological mechanisms of the disease and confirming previous research findings.

Previous studies have suggested that the maximum diameter of the appendix and the presence of fecalith are linked to the failure of non-surgical treatments for uncomplicated appendicitis [29]. Outlet obstruction of the appendix is widely acknowledged as a primary cause of appendicitis [30], with fecalith leading to persistent blockage of the appendix lumen and subsequent increase in intracavitary pressure, promoting the progression of inflammatory processes. Additionally, it is theorized that the diameter of the appendix may enlarge in response to elevated intracavitary pressure, potentially leading to complications such as gangrenous appendicitis and perforation [31]. Therefore, our research suggests that the presence of fecalith and an enlarged appendix diameter are strongly associated with the development of gangrenous appendicitis and the failure of non-surgical interventions.

Abdominal CT scans are commonly utilized for diagnosing acute appendicitis; however, the accuracy for diagnosing gangrenous and perforated appendicitis still requires improvement. In the study conducted by Bolmers et al., it was found that the diagnostic accuracy of CT for acute appendicitis was as high as 94.7%, but its sensitivity for detecting complicated appendicitis was only 35% [32]. Therefore, relying solely on abdominal CT to identify complicated appendicitis presents certain limitations. Additionally, it has been reported that CT may not be sufficient for diagnosing complicated appendicitis, with up to 22% of patients undergoing surgery revealing comorbidities. In response to this, Jens and Stroh ä ker et al. developed a complex appendicitis scoring model based on abdominal CT, which included symptoms, laboratory tests, and CT reports from 1,132 patients [33]. However, this model’s inclusion of symptoms as predictive factors can be subjective and varies significantly between patients.

While abdominal CT is considered the gold standard for diagnosing acute appendicitis, a study by Maxim Avanesov et al. constructed a predictive scoring model for complex appendicitis based on CT reports, achieving a positive predictive value of 94% [34]. While some studies have successfully employed abdominal CT data to develop models for predicting complex appendicitis, demonstrating optimal predictive performance [35], these models have not incorporated clinical factors and laboratory indicators. This suggests a need for further refinement to enhance their predictive capabilities by integrating a broader range of patient information. However, the high cost and radiation exposure associated with enhanced CT limit its utility in identifying complicated appendicitis. Furthermore, all the clinical models or scoring systems from the aforementioned studies were based on data from a single medical center. In contrast, our study utilized data from two different medical centers to develop and validate the model separately, providing significant advantages in clinical applicability.

In our study, we analyzed various clinical characteristics of patients, including age, gender, duration of abdominal pain, history of hypertension and diabetes, history of abdominal surgery, and history of preoperative antibiotic treatment. However, statistical analysis revealed that these factors were not independent predictors of GPA for there was no statistical significance in the results of univariate and multivariate analyses as *P*>0.05.Instead, our scoring model incorporated seven variables - WBC, lymphocytes, D-dimer, blood glucose, albumin, maximum diameter of the appendix, and presence of fecalith - which provided objective and reproducible data for easy utilization.

The evaluation of our scoring model demonstrated its strong discriminative ability in accurately predicting GPA. External validation using patient data from the Fifth Affiliated Hospital of Sun Yat-sen University showed no significant variations in baseline characteristics between the training and testing sets, confirming their suitability as training and validation sets. The nomogram consistently performed well in the verification set, indicating successful validation across diverse populations. This well-calibrated and distinctive nomogram, validated in both training and testing sets, serves as a valuable tool for identifying adult GPA and guiding surgical decision-making in appendicitis patients.

Moreover, the model’s objectivity and reproducibility make it applicable in scenarios where patients may have difficulty providing accurate medical histories or undergoing abdominal examinations due to conditions like impaired consciousness or communication limitations. In our study, we excluded patients over 65 years of age based on the findings of Fugazzola et al. [36], which indicated that appendicitis patients in this age group have a poorer prognosis, with a higher mortality rate, limited efficacy of conservative treatment, and a general recommendation for surgical resection. The objective of our research was to identify high-risk patients with gangrenous or perforated appendicitis, to guide the selection of appropriate treatment plans, and to explore opportunities for non-surgical treatment in certain cases. This focus on a specific population allowed us to investigate the potential for non-surgical management in a group where it might be more feasible. Consequently, further research may be necessary to investigate the risk factors for complicated appendicitis in individuals aged 65 and older.

Despite its strengths, our model acknowledges limitations in result interpretation. Firstly, in our study, we observed differences in data distribution between the training and validation datasets, and the amount of positive event data may have been inadequate when developing the predictive model. Additionally, the retrospective design of the case-control study introduces the potential for selection bias, and the use of data from only two centers may restrict the generalizability of our findings to a broader population at risk of GPA. Further research with larger and more diverse samples is warranted to address these limitations.

## Conclusion

In summary, we have developed a novel scoring model to predict adult GPA by incorporating objective and reproducible variables (WBC, lymphocyte count, D-dimer, blood glucose, albumin levels, maximum diameter of the appendix, and presence of fecalith) obtained from laboratory tests and abdominal CT scans. These variables were carefully chosen for their objectivity and reliability. Our scoring model exhibits a high level of diagnostic accuracy in identifying GPA, enabling the evaluation of disease severity and the selection of optimal surgical timing. It can be easily implemented in clinical practice using routine laboratory tests and abdominal CT scans in most hospital settings, providing valuable support for healthcare professionals in the management of appendicitis cases.

## Data Availability

All data generated or analyzed during this study are included in this article. Further inquiries can be directed to the corresponding author.

## Abbreviations

ROC: Receiver operating characteristic
CT: Computed tomography
GPA: Gangrenous/perforated appendicitis
UA: Uncomplicated appendicitis
AUC: Area under the curve
BMI: Body mass index
WBC: White blood cell
NLR: Neutrophil to lymphocyte ratio
Plt: Platelet
MPV: Mean platelet volume
PDW: Platelet distribution width
Fg: Fibrinogen
D-D: D-Dimer
PCT: procalcitonin
Tbil: Total bilirubin
Glu: Glucose
Alb: Albumin
